# Prevalence and characteristics of *PIK3CA* mutation in mismatch repair-deficient colorectal cancer

**DOI:** 10.7150/jca.37437

**Published:** 2020-04-06

**Authors:** Weihua Li, Tian Qiu, Lin Dong, Fanshuang Zhang, Lei Guo, Jianming Ying

**Affiliations:** Department of Pathology, National Cancer Center/National Clinical Research Center for Cancer/Cancer Hospital, Chinese Academy of Medical Sciences and Peking Union Medical College, Beijing 100021, China.

**Keywords:** * PIK3CA* mutation, MMR, colorectal cancer, *RAS/BRAF* mutations, next-generation sequencing

## Abstract

**Background**: Chromosomal instability (CIN) and microsatellite instability (MSI) account for the major causes of colorectal cancer (CRC). As an important component of the CIN pathway, *PIK3CA* mutation is a negative prognostic factor in CRC. However, the relationship between *PIK3CA* mutation and mismatch repair (MMR) status has not been well clarified.

**Methods**: MMR status was determined by immunohistochemical assay. *KRAS*, *NRAS*, *BRAF*, *PIK3CA* and *TP53* mutations were comparatively analyzed in 424 MMR-proficient (pMMR) and 104 MMR-deficient (dMMR) CRC tumors using next-generation sequencing (NGS).

**Results**: *PIK3CA* mutation was more commonly mutated in dMMR tumors. *PIK3CA* mutation less commonly coexisted with *KRAS/NRAS/BRAF* and *TP53* mutations, but more likely coexisted with *HER2* and *PTCH1* mutations in dMMR tumors compared with pMMR tumors. In tumors with concurrent *RAS/BRAF* and *PIK3CA* mutations, *PIK3CA* and *RAS/BRA*F mutant allele frequencies (MAFs) were highly concordant in dMMR tumors, whereas *PIK3CA* MAFs were significantly lower than the corresponding *RAS/BRAF* MAFs in pMMR tumors, implying that *PIK3CA* mutation may occur in the early stage of dMMR CRC.

**Conclusions**: The molecular pathogenesis is different between dMMR and pMMR tumors with *PIK3CA* mutation in CRC. *PIK3CA* mutation may act as a clonally dominant truncal mutation in dMMR CRC. Thus, combination of *PIK3CA* mutation and MMR status might determine a specific group of CRC to select treatment or elevate prognosis.

## Introduction

Colorectal cancer (CRC) is one of the most common malignancies in the world 1, and ranks as the fifth cause of cancer-related death in China 2. It is a heterogenous disease evolving from diverse genetic pathways, which attribute to tumor development and progression 3. Therefore, uncovering the molecular alterations of CRC may be helpful to develop potential new approaches for the diagnosis and treatment.

Chromosomal instability (CIN) and microsatellite instability (MSI) are two crucial pathways in CRC pathogenesis 4. MSI is a hypermutable phenotype at the genomic level that is caused by deficient DNA mismatch repair (dMMR) mainly because of germline mutations (Lynch syndrome) or hypermethylation of MMR genes 5. MMR status can be determined by immunohistochemical assay (IHC). The dMMR tumors show loss of expression in MLH1, MSH2, MSH6 or PMS2 protein, whereas the pMMR tumors have intact expression of all four MMR proteins. Studies have found that dMMR CRC cases exert some distinct differences in clinicopathologic features compared with pMMR CRC cases, such as preference of proximal colon, mucinous or signet ring differentiation, and a favorable prognosis 6. Moreover, advanced dMMR/MSI-H CRC patients may benefit from immunotherapy, such as anti-PD-1 therapies 7.

*PIK3CA*, which encodes the catalytic p110-alpha subunit of PI3K and thus regulates PIK3CA/AKT pathway downstream of EGFR, has been described to be mutated in 10%-20% of CRC patients. More than 80% of *PIK3CA* mutations occur in the helical and kinase domains 8. Basic studies have reported that mutation in *PIK3CA* can accelerate tumor progression, usually alongside with* KRAS/BRAF* mutations 9, 10. Clinical studies have shown that *PIK3CA* mutation may be a biomarker for resistant to anti-EGFR therapy of CRC 11. Moreover, *PIK3CA*-mutant CRC patients may benefit from adjuvant aspirin therapy or PI3K inhibitor treatment 12, 13. Therefore, *PIK3CA* mutation plays an important role in CRC treatment.

Recent study has reported that* PIK3CA* mutation is more commonly mutated in the MSI molecular subgroup of gastric cancer 14. However, the relationship between *PIK3CA* mutation and MSI status in CRC patients remain elusive. In this retrospective study, we interrogated 424 pMMR and 104 dMMR CRC tumors by NGS to identify *KRAS*, *NRAS*, *BRAF*, *PIK3CA* and *TP53* mutations. We further investigated the clinical and molecular differences of the *PIK3CA*-mutant tumors stratified by MMR status in Chinese CRC patients.

## Patients and Methods

### Patients and specimens

A total of 528 CRC patients who had undergone surgery at Cancer Hospital, Chinese Academy of Medical Sciences (CAMS) between 2013 and 2018 were enrolled. Among these 528 patients, 104 were dMMR CRC, whereas 424 were pMMR CRC. Clinicopathological characteristics were obtained from the medical records. None of the patients had received neoadjuvant therapy or radiotherapy before surgery. The study had been approved by the Institute Review Board of the Cancer Hospital, CAMS. Informed consents were obtained from all patients, and methods were carried out in accordance with the approved guidelines.

### Immunohistochemical assay (IHC)

MLH1, PMS2, MSH2 and MSH6 expression was determined by IHC. Briefly, the tissue sections were deparaffinized in xylene, and then rehydrated in graded alcohol. After washing in distilled water, all tumor samples were stained in an autostainer (Autostainer Link 48, Dako, Denmark) with the antibodies of MLH1 (ES05, Dako) , MSH2 (FE11, Dako), MSH6 (EP49, Dako) and PMS2 (EP51, Dako), respectively.

### Isolation of genomic DNA

All HE slides were observed by an expert pathologist (Dr J Ying) under the microscope, and formalin-fixed and paraffin-embedded (FFPE) tissue blocks with >20% tumor cellularity were used for further NGS testing. For *PIK3CA*-mutant tumors with dMMR, adjacent normal tissues were also selected to identify germline mutations. Genomic DNA was extracted from the selected blocks using QIAamp DNA FFPE Tissue Kits (Qiagen, Duesseldorf, Germany), following the manufacturer's protocols. Qubit 2.0 Fluorometer (Thermo Fisher Scientific, Carlsbad, CA, USA) was used to determine DNA quantity.

### Amplification-based NGS testing

The amplification-based NGS testing was performed to identify mutations in *KRAS*, *NRAS*, *BRAF*, *PIK3CA* and *TP53*. Briefly, multiplex PCR was performed with 10 ng of genomic DNA, and then adapters were ligated to each PCR product. The amplicon libraries were constructed after purifying with 75% ethanol. The quantity of amplicon libraries was determined using Ion Library Quantification Kit (Thermo Fisher, MA, USA). Each library was diluted to a concentration of 40 pM, and pooled in equal volumes. Template preparation was performed with Ion Chef, and sequencing was carried out on Ion S5 with 520 Chip. Finally, data were generated using the software of Torrent Suite. Variants with coverage depth> 500 and MAF >5% were identified as mutations using the Torrent Variant Caller.

### Hybrid capture-based NGS testing

DNA from *PIK3CA*-mutant tumors was further interrogated using a capture-based targeted sequencing panel (Burning Rock Biotech, Guangzhou, China) to parallelly profile somatic mutations of 33 cancer-related genes and MSI status. DNA from adjacent normal tissues was also tested to determine germline mutations. Briefly, fragment genomic DNA was obtained by sonication (M220 Focused- Ultrasonicator, Covaris, Woburn, Massachusetts, USA). PCR amplification was performed after the adaptors were added on both ends. After purification with 75% ethanol, the PCR products were hybridized with the capture probes, and enrichmented with beads. Moreover, PCR amplification was performed to get the libraries. All the indexed libraries were mixed with proper concentration, and were then sequenced on Nextseq N500 (Illumina, San Diego, CA). Sequence data were generated and analyzed by GATK 3.2. Variants with coverage depth> 500 and MAF >5% were identified as mutations.

### Statistical analysis

Molecular and clinicopathological differences between pMMR and dMMR cases were investigated by Chi-square test or Fisher's exact test. The relationship between concurrent *PIK3CA* and *RAS/BRAF* MAFs was determined by paired Student's t-test. All analyses were performed with SPSS 18.0 Software. Statistically significance was identified when a two-sided P-value was less than 0.05.

## Results

### Patient characteristics

**Figure 1:**
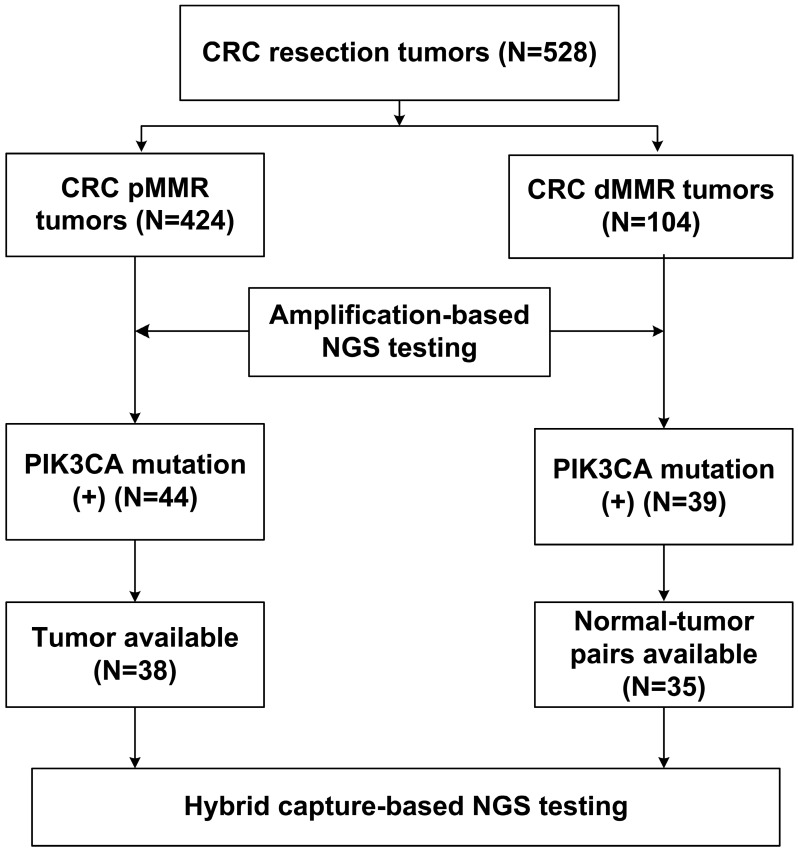
Flow chart of the pMMR and dMMR CRC samples subjected to NGS testing.

With respect to the MMR status, a total of 528 CRC resection cases were classified into 424 pMMR cases and 104 dMMR cases. The clinicopathological characteristics were listed in Table 1, based on the MMR status. The results showed that dMMR cases were significantly associated with right colon location (65.4% vs. 20.8%) and reduced lymph node metastasis (23.1% vs. 73.6%) compared with pMMR cases. In addition, lymphovascular invasion (26.0% vs. 48.1%) and cancerous nodes (5.8% vs. 26.7%) were less frequently observed in dMMR cases than pMMR cases. The study population was subjected to NGS- based molecular testing, as summarized in Figure 1.

### Mutation alterations in pMMR and dMMR tumors

**Figure 2:**
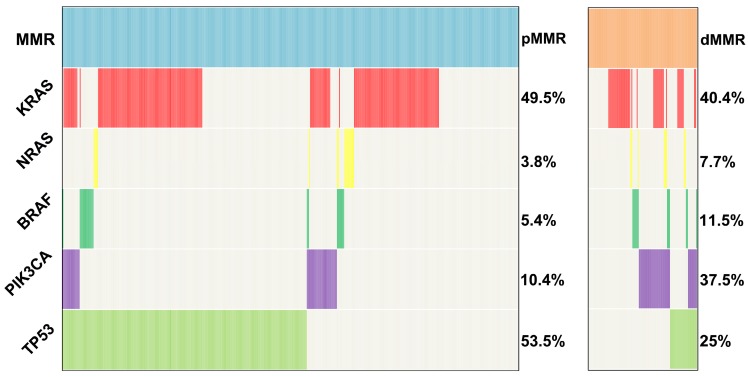
Distribution of *KRAS*,* NRAS*, *BRAF*, *PIK3CA* and *TP53* mutations in 424 pMMR and 104 dMMR CRCs.

*KRAS*, *NRAS*, *BRAF*, *PIK3CA* and *TP53* mutations were tested in 424 pMMR tumors and 104 dMMR tumors using the amplification-based NGS testing. The results showed that *KRAS*, *NRAS*, *BRAF*, *PIK3CA* and *TP53* mutations were observed in 49.5% (210/424), 3.8% (16/424), 5.4% (23/424), 10.4% (44/424) and 53.5% (227/424) of pMMR tumors, respectively. However, *KRAS*, *NRAS*, *BRAF*, *PIK3CA* and *TP53* mutations were observed in 40.4% (42/104), 7.7% (8/104), 11.5% (12/104), 37.5% (39/104) and 25% (26/104) of dMMR tumors, respectively. *TP53* mutation was more frequently to be observed in pMMR tumors than dMMR tumors (53.5% vs. 25%, P<0.001), whereas *PIK3CA* mutation was more likely to be observed in dMMR tumors compared with pMMR tumors (37.5% vs. 10.4%, P<0.001) (Figure 2). To further validated our conclusion, we also investigated the association of MSI status and *PIK3CA* mutation in TCGA databases. A total of 1611 CRC samples detected by MSKCC were included in the analysis as an independent cohort. Interestingly, we found that *PIK3CA* mutation was also more likely to be observed in dMMR tumors compared with pMMR tumors (26.7% vs. 9.4%, P<0.001).

### Clinicopathologic characteristics of pMMR and dMMR cases with *PIK3CA* mutation

The association of clinicopathologic characteristics and *PIK3CA* mutation was investigated in pMMR and dMMR cases, respectively. In pMMR CRC cases, *PIK3CA* mutation was more frequent in older age (59.1% vs. 42.1%, P=0.032) and right colon cancer (45.5% vs. 17.9%, P<0.001). In dMMR CRC cases, no association of gender, age, tumor site, histological differentiation, pT stage, pN stage, lymphovascular invasion and cancerous node with *PIK3CA* mutation was observed (Table 2).

### Concomitant mutations in *PIK3CA*-mutant tumors

Paired tumor-normal tissues from 35 *PIK3CA*-mutant dMMR patients were subjected to the hybrid capture-based NGS testing to parallelly profile somatic mutations of 33 cancer-related genes, MSI status and germline mutations. Moreover, 38 *PIK3CA*-mutant tumors with pMMR were also tested by the hybrid capture-based NGS testing to determine the somatic mutations of 33 cancer-related genes and MSI status. All 35 dMMR tumors were identified as MSI-H, whereas 38 pMMR tumors were MSS/MSI-L. The concordance rate was 100% between NGS-MSI and IHC testing.

**Figure 3:**
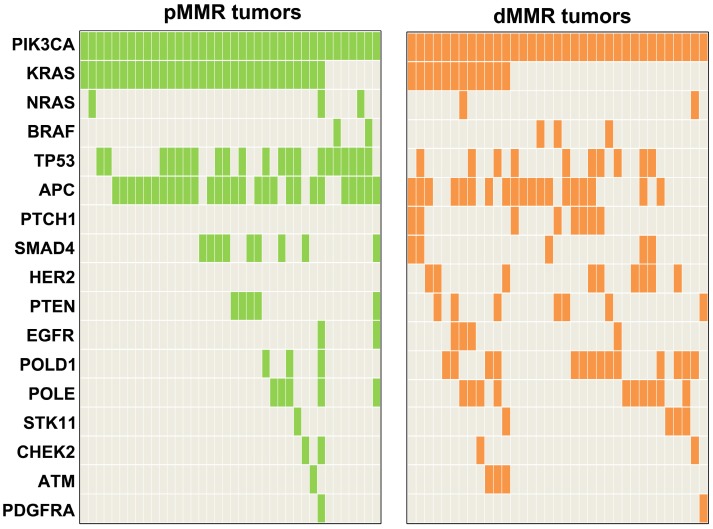
Mutation profiling of *PIK3CA*-mutant tumors by MMR status.

**Figure 4:**
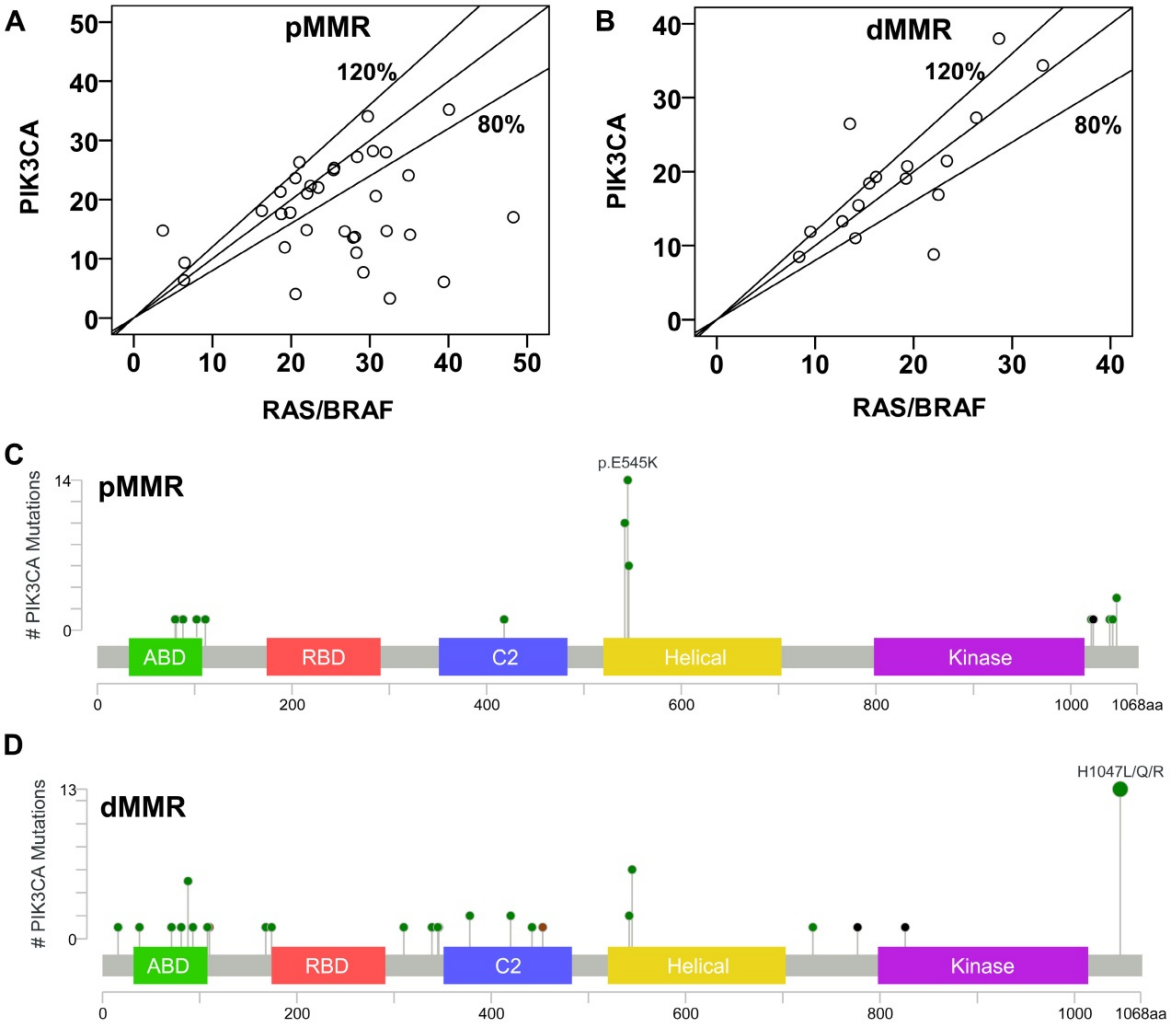
** Molecular characteristics of *PIK3CA*-mutant tumors. (A)** Correlation of MAFs in pMMR tumors with concurrent *PIK3CA* and *RAS/BRAF* mutations. **(B)** Correlation of MAFs in dMMR tumors with concurrent *PIK3CA* and *RAS/BRAF* mutations. **(C) and (D)** The subtype mutations of *PIK3CA* in (C) pMMR tumors and (D) dMMR tumors. Mutations were plotted using the cBioPortal visualization engine.

Concomitant mutations were compared between pMMR and dMMR tumors with *PIK3CA* mutation. The results showed that *PIK3CA* mutation was more commonly to coexist with *KRAS/NRAS/ BRAF* (pMMR vs. dMMR, 89.5% vs. 45.7%, P<0.001) and* TP53* mutations (pMMR vs. dMMR, 55.3% vs. 28.6%, P=0.021) in pMMR tumors, but was more likely to coexist with *HER2* (pMMR vs. dMMR, 0% vs. 25.7%, P<0.001) and *PTCH1* mutations (pMMR vs. dMMR, 0% vs. 22.9%, P=0.006) in dMMR tumors (Figure 3). Moreover, we found that the ratio of *PIK3CA*/*RAS* or *BRAF* MAFs was below 80% in 18.8% (3/16) of dMMR tumors (Figure 4B), but in 52.9% (18/34) of pMMR tumors (P=0.022) (Figure 4A). There was no statistically significant difference between *PIK3CA* (mean ± SD, 19.4 ± 8.6; 95% CI, 14.9-24.0) and concurrent *RAS/BRAF* MAFs (mean ± SD, 18.7 ± 6.9; 95% CI, 15.0-22.4, P=0.612) in dMMR tumors. However, *PIK3CA* MAFs (mean ± SD, 18.1 ± 8.2; 95% CI, 15.2-20.9) were significantly lower than *RAS/BRAF* MAFs (mean ± SD, 26.0 ± 10.4; 95% CI, 22.4-30.0, P = 0.001) in pMMR tumors.

Further, double somatic *PIK3CA* mutations were observed in 37.1% (13/35) of dMMR tumors, which were significantly higher than those in pMMR tumors (4/38, 10.5%, P=0.007) (Table S1). The distribution of* PIK3CA* mutations was shown in Figure 4C and 4D. The results showed that 35 of 38 *PIK3CA* mutations located in exon 9 and 20 in pMMR tumors, whereas 21 of 35 *PIK3CA* mutations located in exon 9 and 20 in dMMR tumors (92.1% vs. 60%, P=0.001). Moreover, *PIK3CA* exon 9 mutation was more common in pMMR tumors, whereas *PIK3CA* exon 20 mutation was more frequent in dMMR tumors (P<0.001). However, no predilection of concurrent *RAS/BRAF* and *PIK3CA* exon 9 mutations was observed in dMMR (3/8, 37.5% vs. 6/13, 46.2%; P=0.948) or pMMR tumors (26/30, 86.7% vs. 6/6, 100%, P=0.813), as compared to concurrent *RAS/BRAF* and *PIK3CA* exon 20 mutations.

In 35 dMMR cases with* PIK3CA* mutation, 6 cases (17.1%) were identified as Lynch syndrome. However, no significant difference was observed between these Lynch syndrome cases and non-Lynch syndrome cases in molecular characteristics (data not shown).

## Discussion

*PIK3CA* mutation is the most common alteration in PI3K pathway, which plays a pivotal role in tumor development 15. *PIK3CA* mutation has been revealed to be a poor prognostic factor, and a negative predictive marker of anti-EGFR therapies in CRC 16. In this study, we interrogated 528 Chinese CRC resection samples using NGS, and retrospectively investigated the molecular and clinicopathologic characteristics of *PIK3CA*-mutant dMMR CRCs stratified by MMR status.

It is reported that* PIK3CA* mutation is related to older age, proximal tumors, mucinous histology, and *KRAS* mutation 17, 18. However, these studies do not stratify CRC cases by MMR status, since pMMR and dMMR tumors show distinct differences in clinicopathologic and molecular characteristics. Our previous study has found that intratumor heterogeneity is more likely to occur in *PIK3CA*-mutant tumors compared with *RAS*-mutant tumors 19. However, the effect of MMR status is not considered, and most of the samples we collect are pMMR tumors in the previous study. Here, we found that *PIK3CA* MAFs were lower than the corresponding *RAS/BRAF* MAFs in pMMR tumors with concurrent *RAS/BRAF* and *PIK3CA* mutations, suggesting that *PIK3CA* mutation may usually occur in the later stage of CRC in pMMR tumors. However, there was no statistically significant difference between *PIK3CA* and concurrent *RAS/BRAF* MAFs in dMMR tumors, indicating that *PIK3CA* mutation may acts as a clonally dominant truncal mutation, and intratumor heterogeneity of *PIK3CA* mutation is uncommon in dMMR tumors.

Consistent with previous studies 6, 20, we found that lymph node metastasis, lymphovascular invasion, left colon/rectum location and *TP53* mutation were more prevalent in pMMR tumors compared with dMMR tumors. Some studies have reported that less consistent or no correlations about the association of *PIK3CA* mutation and MMR/MSI status in CRC 17, 21. However, these studies restrict to hotspot mutation analysis of *PIK3CA* exon 9 and 20 in western CRC population. We here screened *PIK3CA* mutation in all exon using NGS, and identified that *PIK3CA* mutation was more frequent in dMMR tumors than pMMR tumors in Chinese CRC patients.

*PIK3CA* mutation frequently coexists with RAS/RAF/MEK/MAPK pathway activation (often because of *RAS* or* BRAF* mutations) in CRC, leading to poor prognosis 21, 22. In our study, we found that concomitant *RAS/BRAF* and *PIK3CA* mutations were less frequently observed in dMMR tumors than pMMR tumors, further support the notion that *PIK3CA* mutation may act its role as a truncal alteration in the progression of dMMR CRCs. Recent study has shown that agents targeting *PIK3CA* mutation demonstrate encouraging preliminary activity in* PIK3CA*-mutant patients. However, concomitant *RAS/BRAF* mutations may be a potential mechanism of resistance to PI3K inhibitors 14, 23. Thus, our findings suggest that *PIK3CA*-mutant dMMR CRC patients may benefit from PI3K inhibitor because of RAS/BRAF independence. It is reported that *PIK3CA* exon 9, but not exon 20 mutation, is related to *RAS* mutation in CRC 24. Therefore, the association of concurrent *RAS/BRAF* mutations and *PIK3CA* exon 9 and 20 mutations were further analyzed, since the high frequency of *PIK3CA* exon 20 mutation in dMMR tumors in our cohort. However, no significant difference of concurrent *RAS/BRAF* mutations was observed between *PIK3CA* exon 9 and 20 mutations, no matter in pMMR or dMMR tumors. These findings indicate that the RAS/BRAF independent *PIK3CA* mutation has no correlation with the distribution of *PIK3CA* exon mutation in dMMR tumors.

There are some limitations in our study. First, although no significant difference of molecular characteristics was observed between the Lynch syndrome cases and non-Lynch syndrome cases in *PIK3CA*-mutant dMMR tumors, the number of Lynch syndrome cases was relatively small. Our findings need to be validated with future larger studies. Second, higher frequency of double *PIK3CA* mutations was observed in dMMR tumors than pMMR tumors, which we had to attribute to as-yet-unknown covariables. Further basic studies are needed to uncover the function of double *PIK3CA* mutations in dMMR CRCs.

In summary, our study demonstrates that *PIK3CA* mutation is more frequent in dMMR tumors than pMMR tumors. Concomitant* PIK3CA* and *RAS/BRAF* mutations are more likely to be observed in pMMR tumors than dMMR tumors. In tumors with concurrent *RAS/BRAF* and *PIK3CA* mutations,* PIK3CA* MAFs are highly concordant with the corresponding *RAS/BRAF* MAFs in dMMR tumors, whereas *PIK3CA* MAFs are significantly lower than the corresponding *RAS/BRAF* MAFs in pMMR tumors. These data suggest that *PIK3CA* mutation may act as a critical player in the development of dMMR CRC. Therapies targeting *PIK3CA* mutation may gain favorable outcomes in dMMR CRC.

## Supplementary Material

Supplementary table.

## Figures and Tables

**Table 1:** Patient characteristics in 528 CRC patients, including 424 pMMR and 104 dMMR patients.

Clinicopathologic characteristics	N	pMMR	dMMR	P	

**Gender**					
Male	320	260 (61.3%)	60 (57.7%)	0.497	
Female	208	164 (38.7%)	44 (42.3%)		
**Age**					
Median (range)	58 (25-83)	58 (26-83)	56 (25-83)		
<60	304	238 (56.1%)	66 (63.5%)	0.175	
≥60	224	186 (43.9%)	38 (36.5%)		
**Tumor site**					
Left colon	157	132 (31.1%)	25 (24.0%)	<0.001	
Right colon	156	88 (20.8%)	68 (65.4%)		
Rectum	215	204 (48.1%)	11 (10.6%)		
**Histological differentiation**					
Well/Moderate	341	274 (64.6%)	67 (64.4%)	0.970	
Poor	187	150 (35.4%)	37 (35.6%)		
**pT stage**					
pT1-2	54	38 (9.0%)	16 (15.4%)	0.053	
pT3-4	474	386 (91.0%)	88 (84.6%)		
**pN stage**					
pN0	192	112 (26.4%)	80 (76.9%)	<0.001	
pN1-2	336	312 (73.6%)	24 (23.1%)		
**Lymphovascular invasion**					
Yes	231	204 (48.1%)	27 (26.0%)	<0.001	
No	297	220 (51.9%)	77 (74.0%)		
**Cancerous node**					
Yes	119	113 (26.7%)	6 (5.8%)	<0.001	
No	409	311 (73.3%)	98 (94.2%)		

**Table 2:** Clinicopathological features of CRC by *PIK3CA* mutation and MMR status.

Clinicopathologic characteristics	pMMR	*PIK3CA*		P	dMMR	*PIK3CA*		P
		Mutation	Wild-type			Mutation	Wild-type	
**Gender**								
Male	260	28 (63.6%)	232 (61.1%)	0.740	60	23 (59.0%)	37 (56.9%)	0.838
Female	164	16 (36.4%)	148 (38.9%)		44	16 (41.0%)	28 (43.1%)	
**Age**								
<60	238	18 (40.9%)	220 (57.9%)	0.032	66	28 (71.8%)	38 (58.5%)	0.172
≥60	186	26 (59.1%)	160 (42.1%)		38	11 (28.2%)	27 (41.5%)	
**Tumor site**								
Left colon	132	8 (18.2%)	124 (32.6%)	<0.001	25	10 (25.6%)	15 (23.1%)	0.412
Right colon	88	20 (45.5%)	68 (17.9%)		68	23 (59.0%)	45 (69.2%)	
Rectum	204	16 (36.4%)	188 (49.5%)		11	6 (15.4%)	5 (7.7%)	
**Histological differentiation**								
Well/Moderate	274	27 (61.4%)	247 (65.0%)	0.633	67	28 (71.8%)	39 (60.0%)	0.224
Poor	150	17 (38.6%)	133 (35.0%)		37	11 (28.2%)	26 (40.0%)	
**pT stage**								
pT1-2	38	3 (6.8%)	35 (9.2%)	0.805	16	7 (17.9%)	9 (13.8%)	0.575
pT3-4	386	41 (93.2%)	345 (90.8%)		88	32 (82.1%)	56 (86.2%)	
**pN stage**								
pN0	112	15 (34.1%)	97 (25.5%)	0.223	80	33 (84.6%)	47 (72.3%)	0.149
pN1-2	312	29 (65.9%)	283 (74.5%)		24	6 (15.4%)	18 (27.7%)	
**Lymphovascular invasion**								
Yes	204	19 (43.2%)	185 (48.7%)	0.489	27	8 (20.5%)	19 (29.2%)	0.326
No	220	25 (56.8%)	195 (51.3%)		77	31 (79.5%)	46 (70.8%)	
**Cancerous node**								
Yes	113	11 (25.0%)	102 (26.8%)	0.794	6	0	6 (9.2%)	0.128
No	311	33 (75.0%)	278 (73.2%)		98	39 (100%)	59 (90.8%)	
