# Supplementary material for: Prevalence and characteristics of PIK3CA mutation in mismatch repair-deficient colorectal cancer
Source: J Cancer. 2020 Apr 6;11(13):3827–33. doi: 10.7150/jca.37437 (PMC7171509; doi:10.7150/jca.37437)
Supplement: Supplementary file 1 — Supplementary table. [file jcav11p3827s1.pdf]

Table S1. Double somatic *PIK3CA* mutations in dMMR and pMMR tumors.

Case	Mutation 1	Exon	Protein change	MAF	Mutation 2	Exon	Protein change	MAF	MMR status
9	PIK3CA	9	p.E542K	16.90%	PIK3CA	2	p.M16T	13.95%	dMMR
11	PIK3CA	9	p.E545D	27.64%	PIK3CA	5	p.V346E	28.26%	dMMR
12	PIK3CA	2	p.R88Q	14.48%					dMMR
13	PIK3CA	2	p.R88*	27.29%					dMMR
16	PIK3CA	2	p.R88Q	21.18%	PIK3CA	6	p.C378R	17.86%	dMMR
17	PIK3CA	3	p.S174A	5.03%					dMMR
25	PIK3CA	5	p.L339F	38.01%	PIK3CA	8	p.A442V	33.65%	dMMR
30	PIK3CA	20	p.H1047R	7.89%					dMMR
31	PIK3CA	9	p.E545A	8.03%					dMMR
33	PIK3CA	20	p.H1047L	11.02%					dMMR
36	PIK3CA	9	p.E545K	4.44%	PIK3CA	2	p.V71I	8.44%	dMMR
38	PIK3CA	9	p.E542K	13.19%					dMMR
40	PIK3CA	2	p.R88Q	20.74%					dMMR
56	PIK3CA	20	p.H1047R	15.15%	PIK3CA	8	p.C420R	18.48%	dMMR
58	PIK3CA	20	p.H1047Q	6.33%	PIK3CA	2	p.R38S	17.06%	dMMR
59	PIK3CA	3	p.P168S	20.00%					dMMR
60	PIK3CA	2	p.E81K	15.53%					dMMR
66	PIK3CA	2	p.E110del	19.29%					dMMR
67	PIK3CA	20	p.H1047R	8.79%					dMMR
68	PIK3CA	20	p.H1047R	21.13%					dMMR
70	PIK3CA	20	p.H1047R	25.12%	PIK3CA	16	p.R777fs	25.55%	dMMR
74	PIK3CA	5	p.N345K	11.89%					dMMR
76	PIK3CA	9	p.E545K	12.28%					dMMR
80	PIK3CA	9	p.E545G	18.43%					dMMR
81	PIK3CA	20	p.H1047R	12.47%					dMMR
84	PIK3CA	20	p.H1047R	14.02%					dMMR
87	PIK3CA	2	p.R88Q	13.30%					dMMR
93	PIK3CA	20	p.H1047R	8.48%	PIK3CA	2	p.R93Q	7.83%	dMMR
95	PIK3CA	8	p.E453del	4.71%	PIK3CA	17	p.N826fs	5.76%	dMMR
98	PIK3CA	15	p.Q731R	15.47%					dMMR
99	PIK3CA	20	p.H1047R	19.08%	PIK3CA	5	p.R310H	12.81%	dMMR
101	PIK3CA	8	p.C420R	13.19%					dMMR
102	PIK3CA	9	p.E545G	26.47%					dMMR
103	PIK3CA	20	p.H1047R	34.38%	PIK3CA	6	p.C378Y	26.57%	dMMR
104	PIK3CA	20	p.H1047R	21.45%	PIK3CA	2	p.R108H	6.95%	dMMR
105	PIK3CA	9	p.E545K	17.04%					pMMR
110	PIK3CA	9	p.E542K	23.63%					pMMR
131	PIK3CA	9	p.E542K	3.29%					pMMR
178	PIK3CA	9	p.E545K	22.32%					pMMR

190	PIK3CA	9	p.E542K	34.05%					pMMR
215	PIK3CA	9	p.E542K	10.99%					pMMR
219	PIK3CA	9	p.E545K	26.30%					pMMR
220	PIK3CA	9	p.E545K	27.21%					pMMR
233	PIK3CA	20	p.H1047R	22.04%					pMMR
234	PIK3CA	9	p.E542K	25.75%					pMMR
245	PIK3CA	20	p.Y1021H	17.60%	PIK3CA	9	p.Q546R	16.59%	pMMR
249	PIK3CA	9	p.Q546K	17.82%					pMMR
250	PIK3CA	9	p.E542K	31.29%					pMMR
251	PIK3CA	9	p.Q546K	25.00%	PIK3CA	2	p.I102F	23.52%	pMMR
256	PIK3CA	9	p.E545K	14.07%					pMMR
257	PIK3CA	9	p.E542K	25.33%					pMMR
258	PIK3CA	9	p.E545K	21.06%					pMMR
311	PIK3CA	9	p.E545K	13.71%					pMMR
324	PIK3CA	9	p.E545K	24.09%					pMMR
333	PIK3CA	20	p.R1023*	21.34%	PIK3CA	2	p.E81K	19.48%	pMMR
356	PIK3CA	9	p.E545K	10.16%					pMMR
378	PIK3CA	9	p.E542K	7.69%					pMMR
389	PIK3CA	9	p.E545K	20.65%					pMMR
397	PIK3CA	9	p.Q546K	14.78%					pMMR
401	PIK3CA	9	p.E545K	11.91%					pMMR
404	PIK3CA	9	p.Q546P	6.08%					pMMR
406	PIK3CA	20	p.H1047R	18.13%					pMMR
411	PIK3CA	20	p.H1047R	9.32%	PIK3CA	20	p.M1040R	9.24%	pMMR
433	PIK3CA	9	p.E545K	14.69%					pMMR
441	PIK3CA	9	p.E542V	13.55%					pMMR
463	PIK3CA	2	p.R88Q	28.21%					pMMR
478	PIK3CA	9	p.E542K	27.99%					pMMR
481	PIK3CA	8	p.E418K	20.61%					pMMR
489	PIK3CA	2	p.K111E	14.65%					pMMR
501	PIK3CA	9	p.E545K	14.87%					pMMR
505	PIK3CA	20	p.M1043I	35.19%					pMMR
506	PIK3CA	9	p.E545K	4.03%					pMMR
512	PIK3CA	9	p.Q546E	6.37%					pMMR
